# Early-life differences in the gut microbiota composition and functionality of infants at elevated likelihood of developing autism spectrum disorder

**DOI:** 10.1038/s41398-023-02556-6

**Published:** 2023-07-13

**Authors:** Simone Zuffa, Patrick Schimmel, Ayoze Gonzalez-Santana, Clara Belzer, Jan Knol, Sven Bölte, Terje Falck-Ytter, Hans Forssberg, Jonathan Swann, Rochellys Diaz Heijtz

**Affiliations:** 1 Department of Metabolism, Digestion and Reproduction, Faculty of Medicine, Imperial College London, London, SW7 2AZ UK; 2 Laboratory of Microbiology, Wageningen University, Wageningen, the Netherlands; 3 Department of Neuroscience, Karolinska Institutet, 171 77 Stockholm, Sweden; 4 Danone Nutricia Research, Uppsalalaan 12, 3584 CT Utrecht, the Netherlands; 5 Center of Neurodevelopmental Disorders (KIND), Centre for Psychiatry Research; Department of Women’s and Children’s Health, Karolinska Institutet & Stockholm Health Care Services, Region Stockholm, Stockholm, Sweden; 6 Child and Adolescent Psychiatry, Stockholm Health Care Services, Region Stockholm, Stockholm, Sweden; 7 Curtin Autism Research Group, Curtin School of Allied Health, Curtin University, Perth, Western Australia Australia; 8 Development and Neurodiversity Lab, Department of Psychology, Uppsala University, 751 42 Uppsala, Sweden; 9 Department of Women’s & Children’s Health, Karolinska Institutet, Stockholm, Sweden; 10 School of Human Development and Health, Faculty of Medicine, University of Southampton, University Road, Southampton, SO17 1BJ UK

**Keywords:** Autism spectrum disorders, Neuroscience

## Abstract

Evidence from cross-sectional human studies, and preliminary microbial-based intervention studies, have implicated the microbiota-gut-brain axis in the neurobiology of autism spectrum disorder (ASD). Using a prospective longitudinal study design, we investigated the developmental profile of the fecal microbiota and metabolome in infants with (*n* = 16) and without (*n* = 19) a family history of ASD across the first 36 months of life. In addition, the general developmental levels of infants were evaluated using the Mullen Scales of Early Learning (MSEL) test at 5 and 36 months of age, and with ADOS-2 at 36 months of age. At 5 months of age, infants at elevated-likelihood of ASD (EL) harbored less *Bifidobacterium* and more *Clostridium* and *Klebsiella* species compared to the low-likelihood infants (LL). Untargeted metabolic profiling highlighted that LL infants excreted a greater amount of fecal γ-aminobutyric acid (GABA) at 5 months, which progressively declined with age. Similar age-dependent patterns were not observed in the EL group, with GABA being consistently low across all timepoints. Integrated microbiome-metabolome analysis showed a positive correlation between GABA and *Bifidobacterium* species and negative associations with *Clostridium* species. In vitro experiments supported these observations demonstrating that bifidobacteria can produce GABA while clostridia can consume it. At the behavioral level, there were no significant differences between the EL and LL groups at 5 months. However, at 36 months of age, the EL group had significantly lower MSEL and ADOS-2 scores compared to the LL group. Taken together, the present results reveal early life alterations in gut microbiota composition and functionality in infants at elevated-likelihood of ASD. These changes occur before any behavioral impairments can be detected, supporting a possible role for the gut microbiota in emerging behavioral variability later in life.

## Introduction

Autism Spectrum Disorder (ASD) is a persistent early-onset neurodevelopmental condition, defined by the presence of social communication and interaction challenges in conjunction with restricted, repetitive behaviors and atypical sensory processing [1]. The worldwide prevalence rate of ASD is currently estimated to be 1% [2]. Many individuals with ASD experience gastrointestinal (GI) and immune dysfunction [3–5], as well as a range of co-occurring somatic and psychiatric conditions including sleep disorders, epilepsy, and anxiety [1]. ASD is a heterogenous and multifactorial behavioral condition, involving genetic susceptibility, environmental risk factors, and gene-environmental interactions [6]. One such environmental risk factor is the gut microbiota (the trillions of microorganisms that colonize our GI tract), which plays a critical role in host physiology and health [7]. The gut microbiota is now recognized as an important modulator of brain and behavior, including the development and function of social brain networks [8–10], representing a new potential target for intervention in neurodevelopmental conditions such as ASD.

A potential link between the gut microbiota and ASD was suggested almost two decades ago, by observations showing that oral vancomycin treatment resulted in a short-term benefit in a small group of children with regressive-onset autism [11] and that children with ASD presented alterations in the gut microbiota composition (e.g., increased abundance of *Clostridium* species) [12–14]. Subsequently, several studies have shown that GI symptoms, such as abdominal pain, diarrhea, constipation, and flatulence, are more common in children with ASD than their neurotypically developing peers [15] and are positively associated with the severity of behavioral problems (irritability, aggressive behavior, and repetitive behaviors) [16–18]. Multiple cross-sectional studies have also reported an altered gut microbiota composition in children and adolescents with ASD, with lower gut bacterial diversity and an underrepresentation of potentially beneficial bacteria (e.g., *Bifidobacterium* species) [19–22]. However, a clear consensus regarding the specific bacterial taxa or magnitude of changes associated with ASD is lacking. Indeed, a recent multi-omics analysis has highlighted the limitations of cross-sectional cohort studies, thus advocating for longitudinal multi-omic studies in combination with comprehensive patient metadata to advance our understanding of the role that the gut microbiome plays in ASD [23]. At the metabolic level, several neuroactive gut-bacterial-derived metabolites such as 4-ethylphenyl sulfate, *p*-cresyl sulfate, and other structurally related phenolic molecules, have been found to be elevated in a subset of children with ASD [24, 25]. The causal potential of the microbiota in ASD has been suggested from murine studies transferring the fecal gut microbiota from autistic individuals into germ-free mice, resulting in behavioral and molecular changes relevant to this condition [26]. Furthermore, microbial-focused intervention studies like fecal microbiota transplantation (FMT) and novel therapeutic agents that prevents the absorption of microbial metabolites from the GI tract, such as AB-2004 have shown positive preliminary results in ameliorating both autistic traits and GI symptoms [27–29].

Yap and colleagues have recently challenged the notion that the gut microbiota is a key contributing factor in the etiology of ASD, providing evidence that ASD-associated gut microbiota changes can be attributed to low diet diversity [30]. It is indeed well-documented that autistic children often exhibit selective eating patterns [31], and that they are more frequently exposed to antibiotics during the first years of life [32, 33], which can affect the composition of the gut microbiota. However, it remains unclear whether the gut microbiota contributes to, or modifies the likelihood of ASD onset before any dietary changes occur. Emerging evidence suggests that the development of the infant gut microbiota is influenced by host genetics, but that this effect is subtle and affects only certain taxa (e.g., *Veillonella* and *Bacteroides* species) [34]. Prospective longitudinal studies of infants at elevated-likelihood of ASD (i.e., siblings of children with ASD, who have ~20% increased likelihood to develop ASD) can provide insights into the relationship between the early life gut microbiota and the development of ASD [35, 36]. In the present study, we applied an integrated shallow shotgun metagenomic sequencing and ^1^H nuclear magnetic resonance (NMR) spectroscopy-based untargeted metabolomics approach to characterize the gut microbiota developmental profile of infants at elevated versus low-likelihood (i.e., infants without a family history of ASD) of from 5 to 36 months of age.

## Materials and methods

### Participants

Infants were recruited from the Early Autism Sweden (EASE) project, an ongoing longitudinal study of infants at elevated-likelihood of ASD. EASE follows younger siblings of children with ASD (elevated-likelihood) and siblings of typically developing children (low-likelihood) from 5 to 36 months of age. Infants at elevated-likelihood (EL) of ASD in EASE have at least one older sibling with a community clinical diagnosis of ASD according to ICD-10 or DSM-5, which was confirmed by inspection of medical records and an interview with parents led by an experienced child psychologist. Infants were recruited through clinical units, advertisement, and the EASE project website. Infants at low-likelihood (LL) of ASD have no familial history of ASD (in first- or second-degree relatives) and at least one typically developing older full sibling. Both groups were primarily from the larger Stockholm area (Sweden). All infants were born full-term (≥ 36 weeks) and did not have any confirmed or suspected medical conditions, including epilepsy, genetic syndromes associated with ASD, or visual/auditory impairments. Potential participants were excluded if they had been exposed to antibiotics. A total of 35 infants met the eligibility criteria and were included in the study, consisting of 19 EL infants (9 females) and 16 LL infants (10 females). All infants were breastfed until at least 6 months of age. An experienced clinical staff assessed the infants developmental level using the Mullen Scales of Early Learning (MSEL) at 5 and 36 months of age [37]. In addition, the Autism Diagnostic Observation Schedule-Second Edition (ADOS-2) was used to assess symptoms of ASD (communication, social interaction, play and restrictive/repetitive behaviors) at 36 months. Informed written consent was obtained from all parents. This study was approved by the Ethics Board in Stockholm and conducted in accordance with the 1964 Declaration of Helsinki.

### Fecal sample collection

Parents collected approximately 200 mg of fecal material from a single infant’s bowel movement at 5, 10, 14, 24, and 36 months of age at their homes. At each time point, parents were guided with both oral and written instructions to collect the fecal material into a sterile collection tube and contact a dedicated research project assistant, who coordinated the immediate transport of the samples to the laboratory. Samples were transported on dry ice within 30 min of collection through the help of a dedicated courier service. Samples were aliquoted into two sterile tubes under a biological safety cabinet for shallow shotgun metagenome sequencing (~30 mg) and ^1^H NMR spectroscopic analysis (~ 50 mg).

### Shallow shotgun metagenome sequencing

DNA extraction and sequencing was carried out by Diversigen® (Minneapolis, USA) using in-house developed, CLIA-approved BoosterShot® protocol for shallow shotgun sequencing. Briefly, MO BIO’s PowerFecal DNA Isolation Kit (Qiagen, Hilden, Germany) automated for high throughput on QIACube, along with 0.1 mm glass bead plates for bead beating, were used for genetic material extraction. Extracted DNA was quantified using Quant-iT™ PicoGreen™ dsDNA Assay Kit (Invitrogen, Carlsbad, CA). Libraries were then prepared using an adapted procedure from the Nextera XT DNA Library Preparation Kit (Illumina, San Diego, CA). Sequencing was carried out on an Illumina NextSeq using single-end 1 × 145 reads with a NextSeq 500/550 High Output v2 kit (Illumina, San Diego, CA). DNA sequences were aligned to a curated database including all representative genomes in RefSeq for bacteria with additional manually curated strains. Alignments were made at 97% identity against all reference genomes. Every input sequence was compared to each reference sequence within the CoreBiome Venti database using fully gapped alignment with BURST. Ties were broken by minimizing the overall number of unique operational taxonomic units (OTUs). For taxonomy assignment, each input sequence was assigned the lowest common ancestor that was consistent across at least 80% of all reference sequences tied for best hit. The number of counts for each OTU was normalized to the OTU’s genome length. OTUs accounting for less than one millionth of all species-level markers and those with less than 0.01% of their unique genome regions covered (and <1% of the whole genome) were discarded.

### Microbiome analysis

A general overview of the infant gut microbiome composition was generated plotting the most abundant genera of each group at the different timepoints, after calculating the mean relative abundances. The Shannon diversity index, a measure of alpha diversity, was estimated at genus level at each timepoint using the `DivNet` v 0.4.0 package, which takes into consideration the compositional nature of microbiome data, the co-occurrence of the different taxa, and avoids rarefaction [38]. A compositional data analysis workflow was used to investigate the structure of the data and highlight group differences. Zeroes in the OTU count table were imputed using Bayesian-Multiplicative replacement before applying a center log ratio (CLR) transformation with the `CoDaSeq` v 0.99.6 package. Principal component analysis (PCA), from the `mixOmics` v 6.22.0 package, was used to performed dimensionality reduction and the adonis function from the `vegan` v 2.6-4 package was used to perform permutational multivariate analysis of variance (PERMANOVA) on the calculated Aitchison distances. Differential abundance analysis was performed using the `ALDEx2` v 1.30.0 package. ALDEx2 generates distribution probabilities for each analyzed taxon and converts them into distribution of log ratios to account for the compositional nature of microbiome data [39]. Differential abundance is calculated using the Wilcoxon rank-sum test, together with the standardized effect sizes. Obtained p values were corrected for multiple comparisons using Benjamini-Hochberg (BH) correction and only taxa with adjusted *p* values < 0.25 were considered significant and retained for visualization.

### ^1^H NMR spectroscopy

Fecal samples were defrosted on ice and combined with ten 1 mm diameter zirconia beads (BioSpec Products, US) and 700 µL of demineralized water. Samples were homogenized using a Precellys 24 homogenizer (Bertin Instruments, FR) with 2 × 6500 rpm in a 2-min program (2 × 40 s homogenization, with a 20 s interval). Homogenized samples were centrifuged at 10,000 *g* for 20 min at 4 °C. Fecal water (supernatant) was collected from each tube and 630 µL was transferred to a new 1.5 mL Eppendorf. Each tube was supplemented with 70 µL of phosphate buffer (1.5 M KH_2_PO_4_, 2 mM NaN_3_, 1% TSP solution, pH 7.4) and vortexed for ~ 30 s. Samples were centrifuged at 10,000 *g* for 5 min at 4 °C and 600 µL was transferred to 5 mm NMR tubes. Samples were loaded into a refrigerated Bruker SampleJet robot on a 600 MHz UltraShield spectrometer (Bruker Biospin, Karlsruhe, Germany). A standard one-dimensional pulse sequence with saturation of the water resonance was applied (RD-90°-t1-90°-tm-90°-acquire FID, with RD set at 2 s and tm at 100 ms) at 300 K. For each spectrum 8 dummy scans, followed by 64 scans with 32 K data points and a spectral width of 20,000 Hz were acquired.

### ^1^H NMR data processing and analysis

Spectra were manually corrected for phase and baseline distortion to the TSP singlet (δ 0.00) before data acquisition on TOPSPIN 3.2 (Bruker, Germany). The obtained spectra were digitized in MATLAB 2019b using IMPaCTS. TSP (-0.2 to 0.2 ppm) and water (4.7 to 4.9 ppm) regions were removed from the spectra. Peaks were manually checked and aligned using recursive sample-wise peak alignment (RSPA). Spectra were normalized using a probabilistic quotient normalization approach to account for potential dilution factors. PCA analysis with pareto scaling was used for preliminary unsupervised analysis to identify possible outliers. Four samples were categorized as outliers and excluded due to spectral anomalies, most likely derived from issues during spectral acquisition or sample preparation. Pair-wise supervised analysis between groups on full spectra was performed with projection to latent structures-discriminant analysis (PLS-DA) and univariate scaling in MATLAB. Using in-house databases, Chenomx 8.4 (Chenomx Inc, Edmonton, Canada) and HMDB (http://www.hmdb.ca/), all identified peaks were annotated. STOCSY was used to identify peaks belonging to same metabolite (Pearson correlation >0.8). Representative peaks from the different metabolites were integrated and exported for further analysis.

Integrated peaks from ^1^H NMR spectra were log10 transformed. PCA was used to observe the overall structure of the data and sparse PLS-DA from the ‘mixOmics’ package was used to extract the most relevant features that were responsible for differentiating the two groups at the different timepoints. Model performance was evaluated using the perf function of the ‘mixOmics’ package with leave-one-out (loo) cross-validation, given the small sample size. A variable importance projection score (VIP) > 1 was used as a cut-off to identify the most relevant metabolites influencing group separation.

### Integration omics data

Spearman correlations between and within the two omics blocks (microbiome and metabolome data) were calculated using the ‘Hmisc’ v 4.8-0 package. *P* values obtained from multiple comparisons (>250,000) were then corrected for false discovery rate using BH and only correlations with adjusted *p* values < 0.25 were kept in the network. To improve visualization, a subset network was generated retaining only bacterial species directly correlating with GABA or with GABA-correlated metabolites and removing edges within each omics block. The final microbiome-metabolic network was built using the ‘ggraph’ v 2.1.0 and ‘igraph’ v 1.3.5 packages. The network was exported and edited in Cytoscape v 3.8.229.

### Microbial cell culture and metabolomic analysis

Bacteria identified from the fecal microbiome analysis to be associated with GABA abundance were investigated for their potential to influence its presence. This included five different *Bifidobacterium* species, *B. breve* (DSM20091/ATCC15698), *B. bifidum* (DSM20215), *B. longum* subsp. *infantis* (ATCC17930/DSM20218), *B. adolescentis* (DSM20083), and *B. scardovii* (DSM13734), and two *Clostridium* related species, *C. difficile* (DSM1296), and *C. bolteae* (DSM15670) purchased from the DSMZ catalog (Leibniz-Institut DSMZ, Germany). The isolates were screened in anaerobic monocultures (CO_2_/N_2_ (80%/20%) at 1.7 atm) with three different substrates (glutamate, putrescine, and spermidine; 4.5 mM each), which can be converted to GABA, as well as GABA itself (4.5 mM). *Bifidobacterium* and *Clostridium* species were enumerated in MRS, and PYG broth respectively [40, 41]. A modified rich transoligosaccharide propionate medium was used as a base for culturing the above bacterial species anaerobically [41]. The medium consisted of glucose (120 mM, pH 6.2), yeast extract (1 g/L), potassium dihydrogen phosphate (3 g/L), dipotassium hydrogen phosphate (4.8 g/L), magnesium sulfate (0.2 g/L), sodium propionate (15 g/L), and l-cysteine hydrochloride (0.5 g/L). In addition, bacteriological peptone was added to the medium (1 g/L) to accommodate the growth of *Clostridium* species. Serum bottles were supplemented with filter-sterilized vitamin mix and trace metals (100x; originally designed for *Lactobacillus lactis*) [42]. All cultures were incubated for 24 hours at 37 °C. From each culture, 2 mL of bacterial growth medium were collected for ^1^H NMR spectroscopy. From the preliminary results based on growth levels and metabolic activity, *B. breve*, *B. infantis*, *C. difficile*, *C. bolteae* were selected for further analysis. These four strains were, after preculture enumeration, made equivalent in optical density under anaerobic conditions with sterile modified TOS, before transfer to 96-well plates containing 380 µL with a 1:100 inoculation. Media that contained either no supplement or supplemented with GABA was added to the wells. The chosen quadriculture ratios were (*Bifidobacterium* spp.:*Clostridium* spp.): 2:1; 1:1; 1:2. Following 24 h anaerobic incubation [CO_2_/N_2_ (80%/20%) at 1.5 atm], 1 ml of media was collected, centrifuged for 15 min at 10,000 *g* at 4 °C, and then 250 µL of supernatant was transferred into a 1.5 mL Eppendorf. After adding 350 µL of phosphate buffer solution, samples were vortex for ~ 30 s, centrifuged for 5 min at 10,000 *g* at 4 °C, and 600 µL of supernatant was transferred to 5 mm NMR tubes. For the quadricultures, 250 µL was retrieved and mixed with 350 µL of NMR Buffer (1.5 M KH_2_PO_4_, 1 g/L of TSP and 0.13 g/L of NaN_3_), before centrifugation at 10,000 *g* at 4 °C for 5 minutes and transfer to 5 mm NMR tubes. Spectral acquisition, processing, and integration were carried out as described above on a 700 MHz Bruker NMR spectrometer equipped with a cryoprobe. Peaks arising from metabolites of interest within the monoculture and quadriculture screenings were integrated from the NMR spectra and normalized to the respective TSP signals.

### Statistical analysis

All statistical analysis was performed on R version 4.0.2 (R Foundation for Statistical Computing, Vienna, Austria). Fisher’s exact test was used to evaluate differences between categorical variables, whereas Wilcoxon rank-sum test was used for numerical variables if not differently specified.

## Results

### General developmental profiles of infants at elevated- and low-likelihood of ASD

General developmental profiles of infants were evaluated using the MSEL test at 5 and 36 months of age, and with ADOS-2 at 36 months of age. Key confounding perinatal factors such as antibiotic exposure, preterm birth and medical conditions were excluded during enrollment. Additionally, maternal age, family income, and parental education level did not differ between the EL and LL groups (Supplementary Table I).

**Fig. 1:**
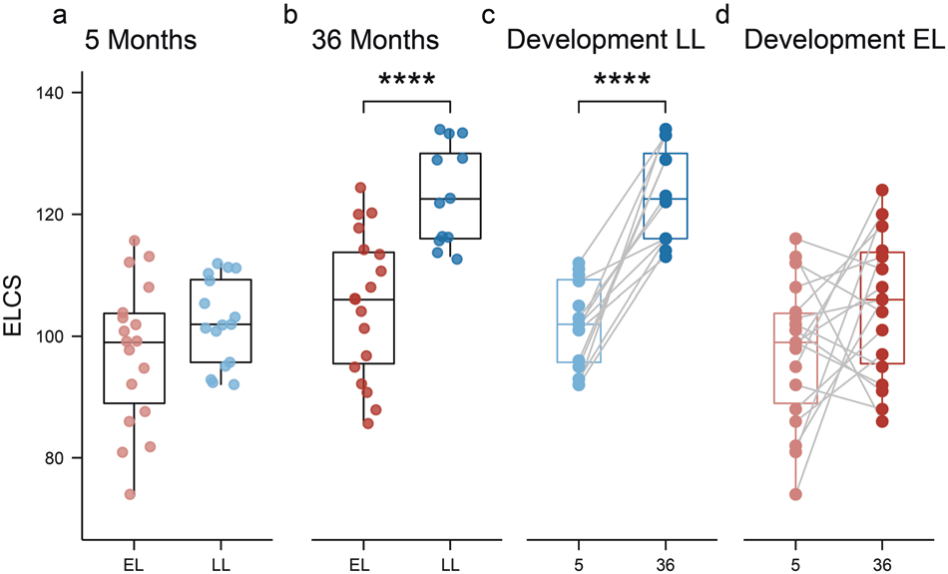
Infants at elevated-likelihood of ASD show lower ELCS compared to low-likelihood infants at 36 months of age. Infants were assessed using the MSEL test at 5- and 36-months of age. **a** At 5 months of age, no significant differences were observed between groups. **b** At 36 months of age, the elevated-likelihood group had a significantly lower ELC scores compared to the low-likelihood (*p* = 0.0002). **c** Infants in the low-likelihood group had a significant increase in their ELC scores between 5 and 36 months, while this developmental change was not observed in the elevated-likelihood group (**d**). Boxplots represent first (lower), median and third (upper) quartile. Wilcoxon test: ***p < 0.001. ELCS Early Learning Composite Scores, EL elevated-likelihood, LL low-likelihood.

At 5 months, the Early Learning Composite Scores (ELCS, which is an age-adjusted standard score) of the MSEL were not significantly different between the EL and LL groups (Fig. 1a). However, at 36 months of age, the LL group had significantly higher ELCS compared to the EL group (Fig. 1b; Wilcoxon test, *p* = 0.0002). Additionally, the LL group had a significant increase in the ELCS between 5 and 36 months of age (Fig. 1c), while this was not the case for the EL group (Fig. 1d). In fact, the ELCS of seven infants in the EL group decreased with age, whereas all infants in the LL group increased with age. At 36 months of age, the overall ADOS-2 scores were significantly higher in the EL group compared to LL group (EL: 6.8 ± 2.484 vs. LL: 4.3 ± 2.720; mean ± SD; Wilcoxon test, *p* < 0.05). However, none of the infants were diagnosed with ASD according to the DSM-5 criteria at 36 months of age.

### Early-life differences in the gut microbiota composition and diversity between infants at elevated- and low-likelihood of ASD

Longitudinal fecal samples from 35 children (19 EL and 16 LL) from 5 to 36 months of age were analyzed by shallow shotgun metagenome sequencing yielding an average of 1,130,684 reads per sample. The number of unique OTUs detected after filtering was 1040. At 5 months, two samples from the LL group were identified as strong outliers and removed from downstream analysis. One sample was dominated by *Clostridium neonatale* (59% of the total relative abundance), while the other sample was dominated by *Klebsiella oxytoca* (44% of the total relative abundance).

**Fig. 2:**
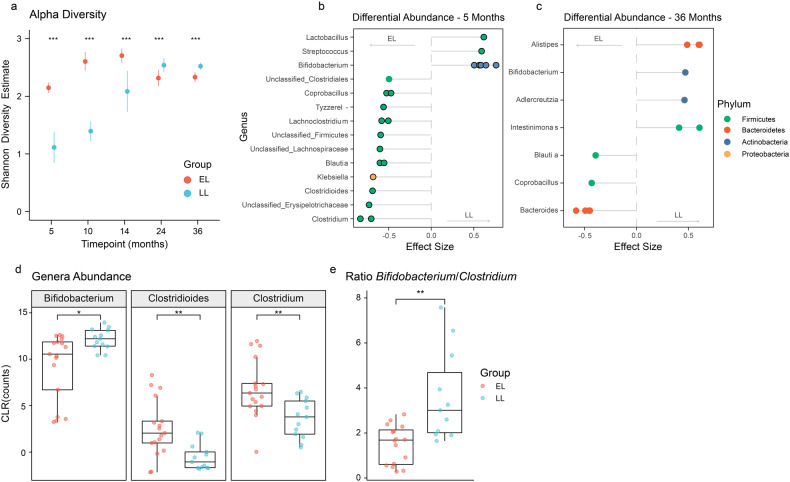
Developmental differences in gut microbial diversity and composition between infants at elevated- and low-likelihood of ASD. **a** Infants at elevated-likelihood of ASD showed higher alpha diversity during the first year of life, but lower diversity thereafter compared to the low-likelihood group. Dots represent mean estimate per group and whiskers show standard errors. **b** At 5 months of age, the low-likelihood group presented more *Bifidobacterium* species (*B. breve, B. Bifidum, B. Longum*, and *B. kashiwanohense*), while the elevated-likelihood group harbored more *Clostridium* related species (*C. clostridioforme, C. neonatale, C. difficile*, and *C. bolteae*), *B. producta, R. gnavus*, and *K. variicola*. Positive effect sizes indicate higher abundance in the low-likelihood group, while negative effect sizes indicate a greater presence of the taxon in the elevated-likelihood group. Displayed OTUs have *p* < 0.05 and a circle stroke indicates if the adjusted *p* value was <0.25. **c** At 36 months of age, none of the OTUs had an adjusted *p* value < 0.25, but several species that were more abundant in the low-likelihood group (*A. senegalensis, A, timonensis*, and *I. butyriciproducens*), as well as different *Bacteroides* species that were more abundant in the elevated-likelihood group had absolute effect sizes >0.5. **d** At 5 months of age, infants at elevated-likelihood of ASD presented significantly less *Bifidobacterium*, and more *Clostridioides* and *Clostridium* compared to infants at low-likelihood of autism. **e** At 5 months of age, infants at elevated-likelihood of autism had a significantly lower *Bifidobacterium*/*Clostridium* ratio compared to the low likelihood one. Boxplots (**d**, **e**) represent first (lower), median and third (upper) quartile. Wilcoxon test: *p < 0.05, **p < 0.01. ELCS Early Learning Composite Scores, EL elevated-likelihood, LL low-likelihood.

The overall gut microbiota composition at genus level of infants from the two different groups during the first 3 years of life is shown in Supplementary Fig. 1. Significant differences were observed in the estimated alpha-diversity (Shannon’s diversity index) between the two groups across all timepoints (DivNet, *p* < 0.001; Fig. 2a), with higher alpha diversity observed in the EL group during the first 14 months of life, but lower diversity thereafter (i.e., between 24 and 36 months). There were distinct differences in the developmental trajectory of alpha-diversity across the two groups. In the LL group, alpha-diversity gradually increased during the first year of life, reaching a plateau at 24 months. In contrast, the EL group showed a pronounced increase in alpha diversity between 5 and 10 months before a moderate decline between 24 and 36 months of age.

### Infants at elevated-likelihood of ASD harbor less *Bifidobacterium* species and more *Clostridium* related species

Unconstrained ordination using PCA of CLR-transformed OTU counts showed a clear time-dependent development (PERMANOVA, R2 = 0.2 and *p* = 0.001) of the infant gut microbiota during the first three years of life (Supplementary Fig. 2). The variance between the individuals was greater during early life (5 and 10 months) than in the later sampling points (PERMDISPER, *p* = 3.59 × 10^−15^). Variation related to sampling age was captured in the first principal component. Inspection of the variables positively and negatively correlated with PC1 (absolute correlation > 0.7) indicated that bacteria belonging to the *Ruminococcaceae*, *Lachnospiraceae, Clostridiaceae*, *Eubacteriaceae* and *Rikenellaceae* families increased with age. Early timepoints were characterized by a higher abundance of taxa belonging to the *Enterobacteriaceae* and *Veillonellaceae* families (Supplementary Table 2).

Further analysis revealed significant differences in the microbial profiles between EL and LL groups at 5 and 36 months of age (PERMANOVA, R2 = 0.07 and *p* = 0.014, R2 = 0.04 and *p* = 0.015 respectively; Supplementary Fig. 3). To identify the OTUs driving the observed differences in the ordination analysis, differential abundance analysis was performed with ALDEx2. OTUs with *p* < 0.05 were retained and effect sizes were plotted. Taxa with adjusted *p* values < 0.25 after Benjamini–Hochberg correction were highlighted and considered significant. Effect sizes between 0.5 and 1 were considered of biological significance for group sample size of 20 [43]. At 5 months, the microbial profiles of infants in the EL group comprised significantly lower amounts of *Bifidobacterium* species (*Bifidobacterium breve*, *Bifidobacterium bifidum*, *Bifidobacterium longum*, *Bifidobacterium kashiwanohense*, and an unclassified species from the *Bifidobacterium* genus) compared to the LL group (Fig. 2b). Additionally, the EL group was characterized by a higher abundance of *Clostridium* related species including *Clostridium clostridioforme*, *Clostridium neonatale*, *Clostridioides difficile* and *Clostridium bolteae*, as well as *Blautia producta*, *Ruminococcus gnavus* and *Klebsiella variicola*, compared to the LL group. Consistent with the species level analysis, the analysis at the genus level showed significant changes in the *Bifidobacterium*, *Lactobacillus*, *Clostridium* and *Clostridioides* genera between the two groups. At 5 months of age, the EL group also had a significantly higher abundance of *Coprobacillus* and *Erysipelatoclostridium*, but a lower abundance of *Alistipes* and *Parabacteroides* compared to the LL group.

Although none of the differentially abundant OTUs at 36 months were significantly different between the two groups after BH correction (*p* adjusted > 0.25), an effect size >0.5 was observed for some of the taxa (Fig. 2c). The EL group presented a lower abundance of *Alistipes senegalensis*, *Alistipes timonensis*, *Intestinimonas butyriciproducens*, and *Bifidobacterium bifidum* compared to the LL group, but a greater abundance of several *Bacteroides* species.

Higher abundance of the *Clostridium* and *Clostridioides* genera and lower presence of *Bifidobacterium* in the EL group was also confirmed through univariate analysis (Wilcoxon test, *p* < 0.05). Boxplots highlighted the presence of 4 outliers within the EL group with a large abundance of *Clostridium* and *Clostridioides* (Fig. 2d). Three of the identified subjects also presented the lowest amount of *Bifidobacterium* compared to the other subjects in the same group. As expected, the *Bifidobacterium*/*Clostridium* ratio was also significantly lower in the EL group compared to the LL group (Fig. 2e).

### Gut metabolome time-dependent development mirrors gut microbiome development

Analysis of the metabolites extracted from the longitudinal fecal samples of infants at elevated- and low-likelihood of ASD show a time-dependent development of the fecal metabolome during the first three years of life (PERMANOVA, R2 = 0.2 and *p* = 0.001; Supplementary Fig. 4). Variance within each timepoint progressively reduced through time. Fecal samples collected at earlier timepoints contained greater amounts of energy-related metabolites like formate, lactate, pyruvate, and metabolites derived from breast milk, including human milk oligosaccharides (HMOs), fructose, and galactose. No differences were noted in HMOs across the study groups, consistent with the parent reports, indicating no variation in breastfeeding between EL and LL infants. Samples collected at later timepoints contained greater amounts of isovalerate, valerate, urocanate, trimethylamine (TMA), and two unknown metabolites (doublet at 0.86 ppm and triplet at 0.85 ppm, both correlated to valerate) tentatively identified as isocaproate and valproate.

### Infants at elevated-likelihood of ASD excrete lower amounts of fecal GABA at 5 months of age

**Fig. 3:**
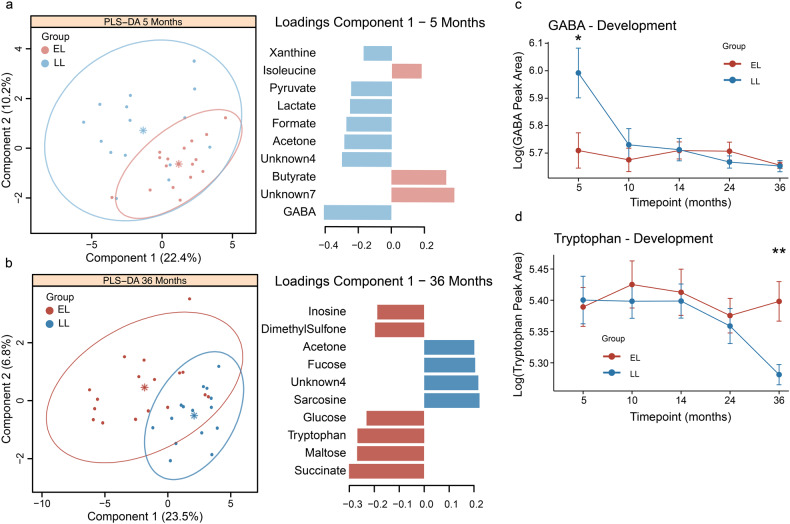
Developmental differences in fecal metabolomes between infants at elevated- and low-likelihood of ASD. Sparse PLS-DA models at 5- and 36-months of age were constructed with integrated metabolites from the full ^1^H NMR spectra. Metabolites with VIP > 1 were retained for the visualization of the loading plots of the first principal component. **a** GABA was identified as the main discriminatory metabolite driving the separation between infants at elevated- and low-likelihood of ASD at 5 months of age. **b** At 36 months, infants at elevated-likelihood of ASD presented more succinate, tryptophan, maltose, and glucose and less sarcosine compared to infants in the low-likelihood group. **c** Developmental changes in GABA and **d** tryptophan concentrations in infants at elevated- and low-likelihood of ASD. Group centroids are represented as * and ellipses represent 95% confidence interval. EL elevated-likelihood, LL low-likelihood.

Differences were observed in the fecal metabolomic profiles between elevated- and low-likelihood infants 5 to 36 months of age. Sparse PLS-DA models were generated with the integrated metabolites, and the loadings for metabolites with VIP > 1 were extracted. At 5 months, infants from the LL group were characterized by a greater presence of GABA and the energy-related metabolites, formate, lactate, and pyruvate, while the EL infants excreted greater amounts of butyrate and isoleucine (Fig. 3a). Univariate analysis confirmed these observations; with GABA abundance significantly lower in EL group compared to LL group (*p* < 0.05; Supplementary Fig. 5). In addition, butyrate appeared to be higher in the EL group, but not statistically significant (Supplementary Fig. 5).

At 36 months, the fecal metabolomes of the two study groups were also found to differ. A greater excretion of succinate, tryptophan, maltose, and glucose was observed in the EL infants whereas the LL infants excreted greater amounts of sarcosine (Fig. 3b). Observations were also confirmed through univariate analysis (Supplementary Fig. 5).

**Fig. 4:**
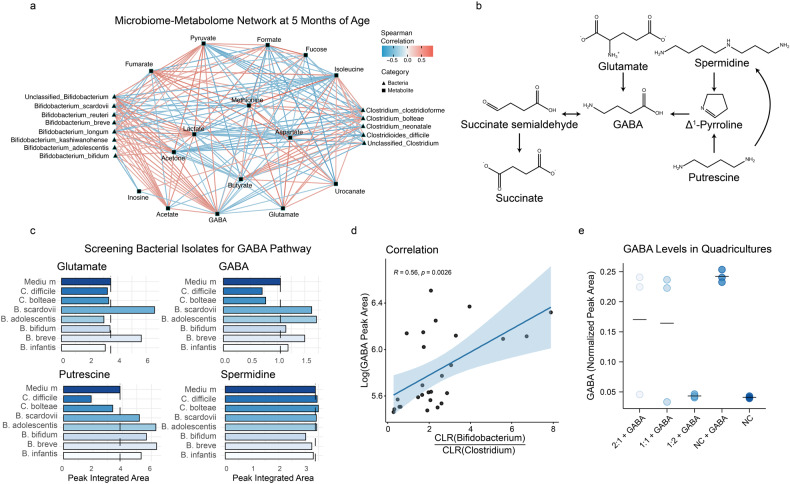
Integration of fecal microbial and metabolic profiles of infants at elevated- and low-likelihood of ASD and in vitro studies. **a** Network constructed on CLR transformed OTUs and log transformed metabolites at 5 months of age using Spearman correlations. The full network was initially generated using all available OTUs and metabolites and correlations with adjusted *p* values < 0.25 were retained (full network available in Supplementary Fig. 6). To improve visualization, only *Bifidobacterium* and *Clostridium* species of interest and their correlated metabolites are displayed. GABA and acetate were positively correlated to the *Bifidobacterium* species of interest, while the *Clostridium* related species correlated with butyrate and glutamate. Red edges indicate positive correlations and blue edges indicate negative correlations. Edge width scaled on the absolute correlation values. **b** GABA can be produced through the GABA shunt, a closed-loop process that converts the α-ketoglutarate from the TCA cycle into glutamate, then GABA and finally into succinate, which re-enter the TCA cycle. GABA can also be produced from polyamines (e.g., spermidine and putrescine). Three different substrates (glutamate, putrescine, and spermidine) that can be converted into GABA were used in the *Bifidobacterium* and *Clostridium* cultures. **c** Bacterial isolates were cultured for 24 h in monocultures with the added substrate. *B. breve* and *B. scardovii* produced glutamate. GABA was produced by *B. breve*, *B. adolescentis* and *B. scardovii*, only when already present in the medium, and consumed by *C. difficile* and *C. bolteae*. Putrescine was consumed by *Clostridium* related species and produced by *Bifidobacterium* species, only when already present in the medium. No interactions with spermidine were observed. **d** A positive correlation was found between *Bifidobacterium*/*Clostridium* ratio and GABA concentration in fecal samples. **e** To determine whether the ratio of *Bifidobacterium* to *Clostridium* species. influenced the abundance of GABA, different growth conditions were investigated in vitro (*Bifidobacterium* spp.: *Clostridium* spp.; 2:1, 1:1, 1:2). GABA abundance was reduced to values comparable to that of the negative control (NC) when a greater proportion (2:1 *Clostridium* spp.: *Bifidobacterium* spp.) of *Clostridium* spp. were present in the cultures. Group means are plotted as line.

A microbiome-metabolome network was constructed using Spearman correlations between CLR-transformed OTUs and log-transformed integrated metabolites at 5 months. Only correlations with adjusted *p* value < 0.25 were retained during the construction of the full network (Supplementary Fig. 6). To simplify visualization, only *Bifidobacterium* and *Clostridium* related species of interest were plotted, together with the significantly correlating metabolites (Fig. 4a). *Bifidobacterium* species showed a strong positive correlation with GABA, acetate, and acetone. Some of the bifidobacteria were also positively associated with pyruvate and formate. *Clostridium* related species were strongly correlated with butyrate and glutamate and negatively correlated with acetone and lactate. Interestingly, GABA was positively correlated with acetate, acetone, lactate, and pyruvate and negatively correlated with glutamate and butyrate.

### Regulation of GABA synthesis by *Bifidobacterium* and *Clostridium* species in vitro

To validate the statistical correlations between GABA and the discriminatory bacterial species, human isolates were grown in the presence of four different metabolites involved in the GABA pathway (Fig. 4b). Specifically, *B. breve, B. bifidum, B. longum subsp. infantis, B. adolescentis, B. scardovii, C. difficile*, and *C. bolteae* were cultured under anaerobic conditions in cell culture medium supplemented with either GABA, glutamate, putrescine, or spermidine.

Supernatants were collected after 24 h and analyzed by ^1^H NMR spectroscopy to assess the production and consumption of metabolites of interest (Fig. 4c). Both *B. breve* and *B. scardovii* produced glutamate when this amino acid was added to the culture medium (Fig. 4c). Consistent with the in vivo correlations, *B. breve*, *B. scardovii*, and *B. adolescentis* produced GABA, but exclusively when this neurotransmitter was added to the media (Fig. 4c), while the *Clostridia related species*. (e.g., *C. difficile* and *C. bolteae*) consumed it. All *Bifidobacterium* spp. produced putrescine, but only if this metabolite was supplemented to the medium. In contrast, *Clostridia* related species consumed putrescine. No interactions with spermidine were observed. Consistent with these in vitro observations, there was a positive correlation between GABA abundance and the Bifidobacterium/Clostridium ratio in the fecal microbiome of infants at 5 months of age (*R* = 0.56, *p* = 0.0026; Fig. 4d).

To assess whether the ratio of *Bifidobacterium* to *Clostridium* species influenced the abundance of GABA in the cultures, three growth conditions were studied (*Bifidobacterium* spp.: *Clostridium* spp.; 2:1, 1:1, 1:2). The species selected after initial experiments were *B. breve*, *B. Infantis*, *C. difficile*, and *C. bolteae*. To maintain GABA abundance around 4.5 mM, a higher initial ratio of *Bifidobacterium* species. was required (2:1; 1:1, Fig. 4e). Although this response was variable, GABA abundance was reduced to values comparable to that of the negative control (NC) when a greater proportion (2:1 *Clostridium* spp.: *Bifidobacterium* spp.) of *Clostridium* species were inoculated (Fig. 4e).

## Discussion

The gut microbiota is increasingly recognized as a modulator of brain development and behavior, but its role in the etiology of common neurodevelopmental conditions such as ASD is poorly understood. Here, we report for the first time early life alterations in the gut microbiota composition and metabolic profile of infants at elevated-likelihood of ASD, during a critical period when the gut microbiota and brain are both undergoing rapid development [44]. Specifically, we found that infants at elevated-likelihood of ASD harbored less *Bifidobacterium* and more *Clostridium* related species at 5 months of age compared to infants at low-likelihood of ASD. This was accompanied by a significantly lower abundance of GABA in fecal samples from infants at elevated-likelihood of ASD. These early differences in the gut microbiota profile were also associated with changes in receptive language and expressive language from 5 to 36 months of age. These findings suggest that the intestinal microbiota of infants at elevated-likelihood of ASD develop differently to those at low-likelihood, with consequences for the bioavailability of GABA in the gut, and its neuro-immune modulatory effects on the host in early life. Although none of the infants were diagnosed with ASD at 3 years of age, more subtle presentations of ASD might still be diagnosed later in life, when everyday functional demands and expectancies increase (e.g., in educational settings) [45]. Indeed, only a minority of ASD cases are diagnosed in Sweden before age 5 [46]. Furthermore, about 28% of infants at elevated likelihood of ASD will also experience a range of neurodevelopmental difficulties, which will not reach the threshold for a clinical diagnosis of ASD [47]. Therefore, it will be important to follow-up this cohort of infants until school age.

The gut microbiota of infants at elevated-likelihood of ASD displayed a greater alpha diversity during the first year of life compared to the microbiota of low-likelihood infants, while the opposite was observed at 2 and 3 years of age. Previous studies have reported inconsistent differences in alpha diversity in infants with ASD compared to neurotypical siblings or healthy controls [12, 48–53]. However, these studies analyzed samples from children between the ages of 1 and 12 years, which is in alignment with the decrease in alpha diversity observed from the second year of life onwards. Differential abundance analysis showed a lower presence of *B. breve*, *B. bifidum*, *B. longum*, and *B. kashiwanohense* species in the elevated-likelihood of ASD group compared to the low-likelihood individuals. Decreased presence of *Bifidobacterium* has been previously observed in autistic children [21, 22]. Members of the genus *Bifidobacterium* (*B. longum subsp. Infantis, B. bifidum, B. breve*, and *B. longum subsp. Longum*) are among the first colonizers of the neonatal gut, and the most abundant taxa in vaginally born and breastfed infants [54]. Bifidobacteria have evolved a series of complex generic pathways to metabolize human milk oligosaccharides (HMOs), which support their growth [55]. The early life dominance of bifidobacteria have been associated with various health benefits, including folate production in the gut, protection against pathogens and development of the immune system, while their reduced levels have been associated with immune and metabolic disorders [56]. Moreover, we also found that infants at elevated-likelihood of ASD presented greater amounts of *C. bolteae*, *C. difficile*, *C. clostridioforme*, *C. neonatale, B. producta, R. gnavus*, and *K. variicola* compared to the low-likelihood group. *Clostridium* species are considered pathobionts and responsible for inflammation when homeostasis is disturbed [57]. Previous studies have shown higher abundance of *Clostridium* spp. (*C. bolteae, C. difficile*, and *C. clostridioforme*) in children with ASD [58–60]. Interestingly, the abundance of *B. producta* at 1 year of age has been associated with adverse social behavior at 3 years of age [61] while *R. gnavus* has been associated with depression [62] and was negatively correlated with cognitive functions in children [63]. Finally, *K. variicola* was found to be higher in pregnant women with gestational diabetes [64], a likelihood factor for ASD development [65]. When aggregated at the genus level, infants at elevated-likelihood of ASD presented less *Alistipes* and *Parabacteroides* and more *Coprobacillus* compared to infants in the low-likelihood group. Decreased abundance of *Alistipes* and *Parabacteroides* has been previously observed in children with ASD [66], while increased presence of *Coprobacillus* has been reported in ASD children with repetitive eating behavior disorders [67].

Metabolic profiling identified GABA as a discriminatory metabolite in feces driving the separation between infants at elevated- and low-likelihood of ASD at 5 months of age. GABA is the main inhibitory neurotransmitter in the adult central nervous system (CNS), and its imbalance has been implicated in different neurological and psychiatric disorders. Early in development, however, GABAergic synaptic transmission is excitatory, and plays an important role in neurodevelopment [68, 69]. Mounting evidence from human neuroimaging, postmortem, and genetic studies indicate an imbalance in excitatory/inhibitory neurotransmission ratio in ASD [70]. Moreover, lower amounts of GABA have also been detected in fecal samples of autistic children [49], suggesting a potential role of the GABA pathway in the neurobiology of ASD. The available evidence indicates that bacterial-derived GABA present in the blood does not enter the brain. Studies using germ-free mice mono-colonized with *Bifidobacterium dentium*, which possesses the enzymatic machinery to produce GABA from glutamate, glutamine, and succinate, have shown an increase in fecal GABA concentrations, but no changes in the brain [71]. This is consistent with the notion that GABA is unable to cross the blood–brain barrier. However, there is evidence that the central GABA pathway can be modulated via the vagus nerve and enteric nervous system. For instance, Bravo et al. demonstrated that ingestion of *Lactobacillus reuteri*, a probiotic with anti-inflammatory properties, can regulate emotional behavior and central GABA receptor expression in mice via the vagus nerve [72]. Moreover, recent findings have implicated GABA producing species such as *Bifidobacterium infantis* in the early life programming of the immune system [73]. Taken together, these observations raise the possibility that bacterial-derived GABA could modulate the central nervous system indirectly via neuronal (e.g., vagus nerve) and immune pathways.

In addition to the observed changes in GABA, infants at elevated-likelihood of ASD tended to excrete higher amounts of butyrate, which has previously been observed in greater concentrations in the stool samples of autistic children [74–76]. Although butyrate is considered a beneficial metabolite in the adult gut, animal studies indicate a dose-dependent effect on brain development and function, with higher doses causing a stress-like response [77]. At 36 months of age, the metabolic composition of the feces from the elevated-likelihood group was characterized by a greater presence of tryptophan and succinate, but lower amounts of sarcosine compared to the low-likelihood group. Previous studies have shown that children with ASD excrete higher amounts of tryptophan in the urine compared to their neurotypical peers [78]. In the gut, tryptophan can be metabolized into several compounds such as the neurotransmitter serotonin (5-HT), kynurenines, tryptamine, and indolic metabolites (under direct or indirect regulation by the gut microbiota). These compounds have been implicated in the communication along the microbiota-gut-brain axis [79]. Sarcosine, also known as *N*-methylglycine, can be converted into glycine, which has a fundamental role in the CNS homeostasis [80]. Interestingly, a previous study noted that fecal microbiota transplant (FMT) intervention in children with ASD improved their GI and ASD symptoms with a concomitant increase in plasma sarcosine [81, 82].

Integration of microbial and metabolic profiles showed a strong positive correlation between the identified *Bifidobacterium* species and GABA. In contrast, *Clostridium* species were correlated with glutamate and butyrate. Glutamate is a direct precursor of GABA, and the negative correlation between these two metabolites suggest an accumulation of this amino acid in the gut due to a lower conversion to GABA, given the reduced presence of bifidobacteria in the elevated-likelihood infants. Collectively, these observations raised the possibility that competition between *Clostridium* and *Bifidobacterium* species may exist and lead to reduced availability of GABA. To test this hypothesis, we used a simplified in vitro model system with different ratios of the identified *Bifidobacterium* and *Clostridium* species in the presence of GABA and its metabolic precursors. Fascinatingly, *B. breve*, *B. scardovii*, and *B. adolescentis* produced GABA when it was added to the medium, while *C. difficile* and *C. bolteae* degraded GABA. These results support the notion that a delicate balance exists between *Bifidobacterium* and *Clostridium* species in the infant gut, with clear consequences for the availability of GABA and its modulatory effects on the infant host.

The general development level of infants was assessed at 5- and 36-months of age using the MSEL. Consistent with previous findings, no significant differences were observed at 5 months of age between the elevated- and low-likelihood of ASD groups [83]. At 36 months, however, infants in the elevated-likelihood group had lower ELCS compared to those in the low-likelihood groups. However, none of the infants in our cohorts develop impairing ASD symptoms, leading to a clinical diagnosis. It is well-known that autistic individuals with good verbal and intellectual abilities can be diagnosed later in development, when daily demands increase. Moreover, other co-occurring mental health issues such as anxiety and depression may not be evident at this early age.

The absence of major perinatal complications in this cohort implicates host genetic risk factors in the observed differences in gut microbiota developmental profiles between the elevated- and low-likelihood groups at 5 months of age. Along this vein, recent studies have shown that many high-confidence risk genes for ASD are also expressed in the developing gut [84], suggesting that genetic variants previously linked to behavioral symptoms in ASD could contribute to host-microbial interactions early in life. In future prospective longitudinal studies of infants at elevated-likelihood of ASD, it will be important to include mother-father-child triads, coupled with the collection of genetic information of infants and their families, mother gut microbiota composition (from pregnancy until the first postnatal year of life), HMO content in breast milk, and relevant clinical outcomes.

In conclusion, the present study revealed distinct gut microbial and metabolic developmental profiles of typically developing infants with and without family history of ASD during the first 3 years of life. These differences were more pronounced at 5 months of age and characterized by lower abundance of beneficial *Bifidobacterium* species and GABA, and by an increased abundance of *Clostridium* related species and butyrate in the fecal samples of infants at elevated-likelihood of ASD. Further mechanistic studies are required to elucidate the impact of these species and metabolites on host development and their possible role in the etiology of ASD and associated comorbidities such as GI problems and anxiety. The supplementation of psychobiotic diet (e.g., prebiotics and probiotics) in early life should be explored as a strategy to promote healthy development of infants at EL of ASD.

## Supplementary information

Supplementary information
