# Supplementary material for: Early-life differences in the gut microbiota composition and functionality of infants at elevated likelihood of developing autism spectrum disorder
Source: Transl Psychiatry. 2023 Jul 13;13:257. doi: 10.1038/s41398-023-02556-6 (PMC10344877; doi:10.1038/s41398-023-02556-6)
Supplement: Supplementary file 1 — Supplementary information [file 41398_2023_2556_MOESM1_ESM.docx]

**Supplementary Information**

**
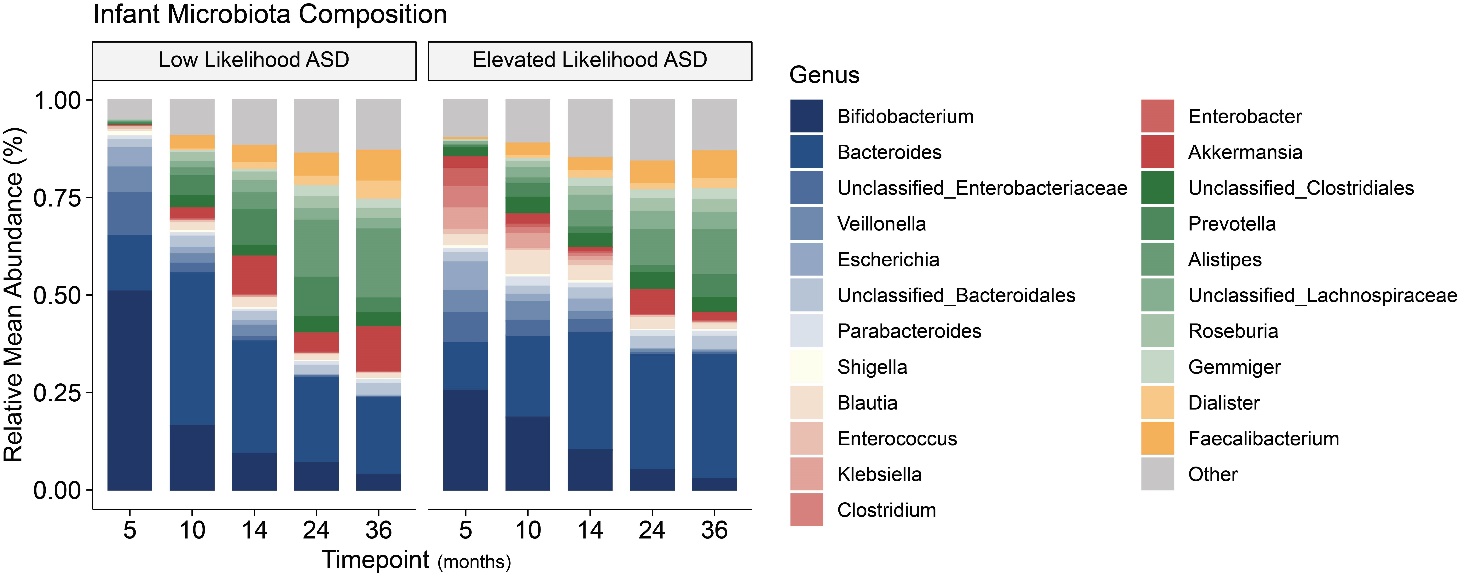
**

**Supplementary Figure 1. Gut microbiota composition level of infants at elevated- and low-likelihood of ASD during the first three years of life**. The relative mean abundance of the top taxa was calculated using the aggregated OUTs at the genus level. The largest differences between the two groups were observed at 5 months of age. The low-likelihood group harbored more *Bifidobacteria* (51% vs 26%), while the elevated-likelihood group had more *Akkermansia* (3% vs 0.003%), *Enterobacter* (5% vs 0.006%), *Clostridium* (5% vs 0.005%) and *Klebsiella* (6% vs 0.004%) species compared to the low-likelihood group.

.

**
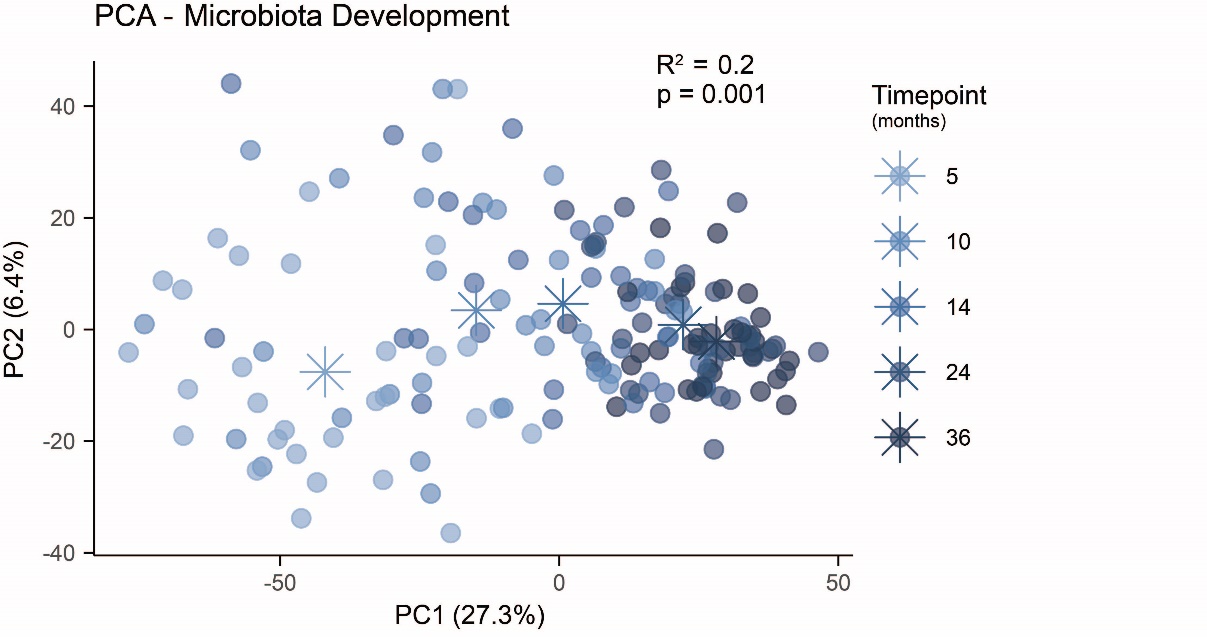
**

**Supplementary Figure 2. Time-dependent changes of the gut microbiota during the first three years of life.** PCA showed a clear time-dependent development of the gut microbiota over the first three years of life, explaining ~ 20% of the total variance observed in the data (PERMANOVA, R2 = 0.2, *p* = 0.001). Variance within time group was greater in early time points compared to later ones (PERMDISPER, *p* = 3.59 x 10-15). PC1 captured the time dependent variation and correlated OTUs showed that early timepoints were characterized by OTUs mainly belonging to the *Enterobacteriaceae* and *Veillonellaceae* families while the later ones by OTUs belonging to the *Ruminococcaceae*, *Lachnospiraceae*, *Clostridiaceae*, *Eubacteriaceae* and *Rikenellaceae* families. Group centroids are represented as *.

**
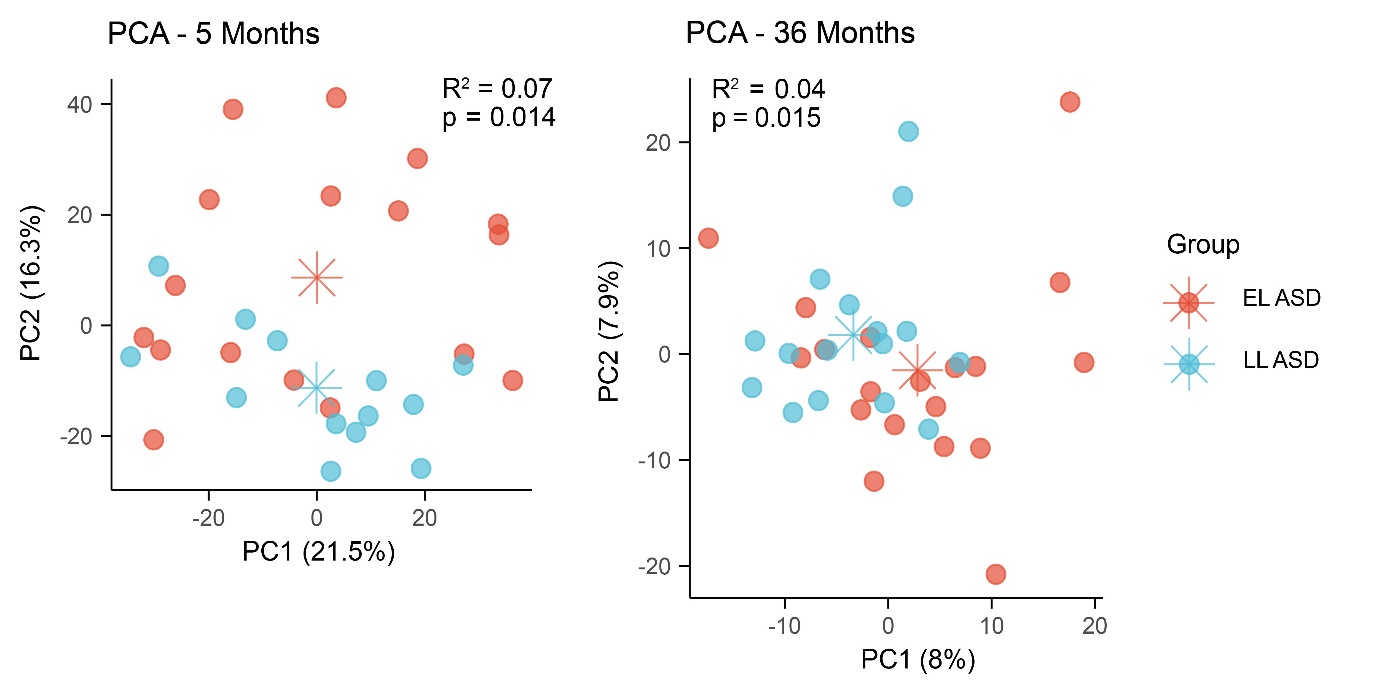
**

**Supplementary Figure 3. The gut microbiota profiles of infants at elevated- and low-likelihood of ASD differs at 5 and 36 months of age.** PCA of CLR transformed OTUs and PERMANOVA on calculated Aitchison distances showed significant differences between the two groups at 5 and 36 months (R2 = 0.07, p = 0.014 and R2 = 0.04, p = 0.015 respectively). Group centroids are represented as *


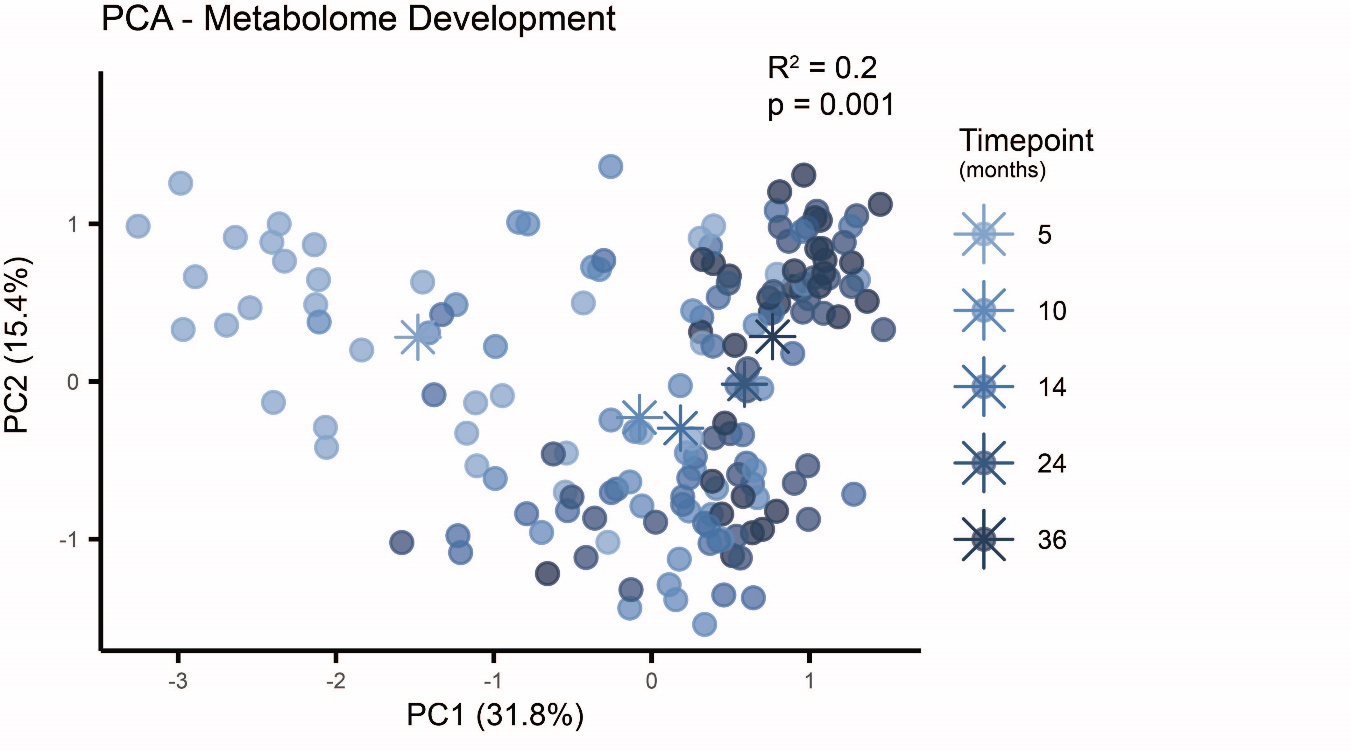


**Supplementary Figure 4. Gut metabolome time-dependent development during the first three years of life.** PCA showed a time-dependent development of the gut metabolome, which mirrored the one observed in the microbial profiles, explaining also in this case ~ 20% of the total variance observed in the data (PERMANOVA, R2 = 0.2 and p = 0.001). Variance at 5 month of age was greater compared to later timepoints (PERMDISPER, *p* = 3.98 x 10^-13^). Early timepoints were characterized by energy and breast milk related metabolites, such as formate, lactate, pyruvate, fructose, galactose, and HMOs, while later timepoints by dietary animal products derivates, like TMA, urocanate and valerate. Group centroids are represented as *.


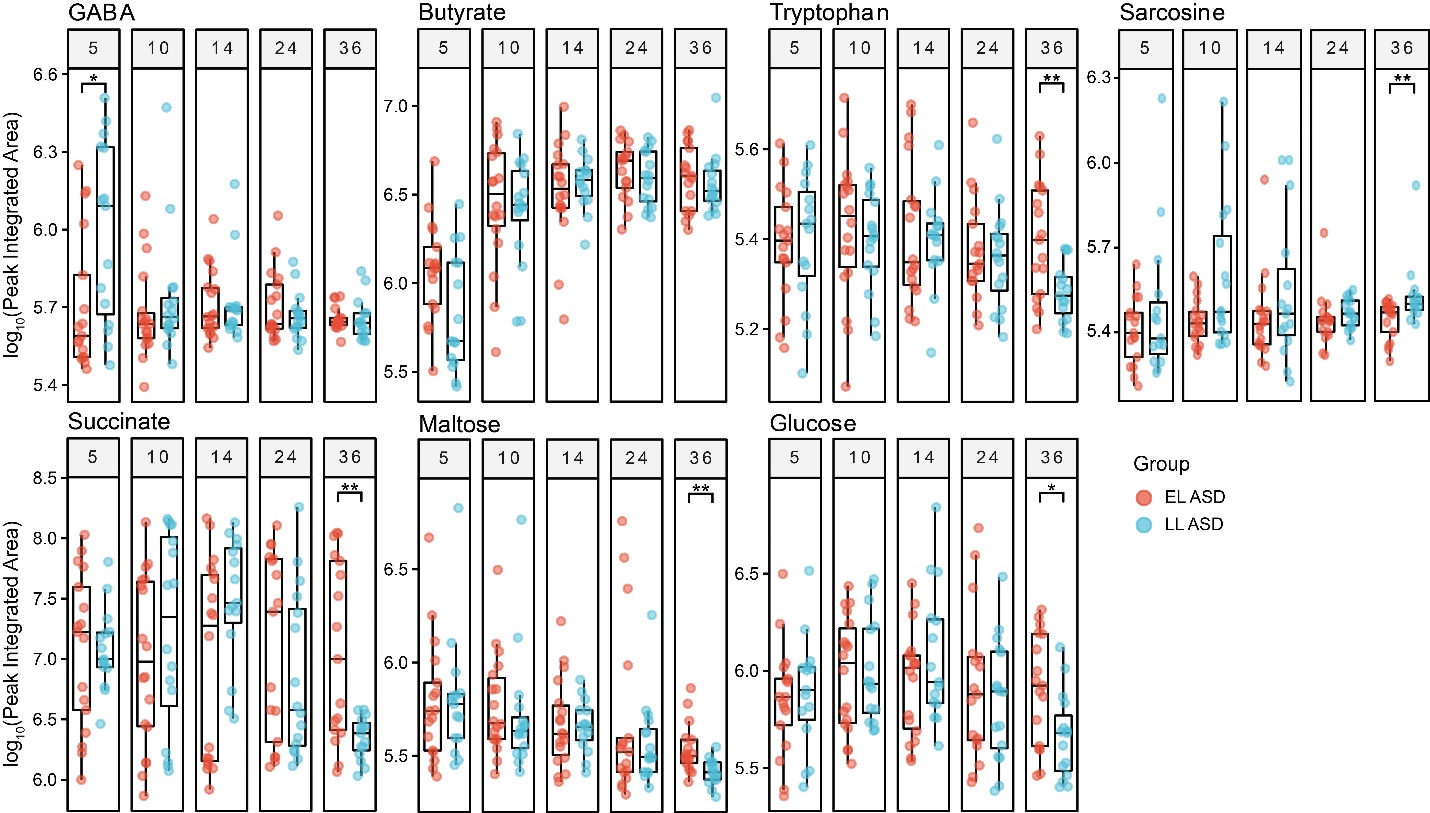


**Supplementary Figure 5. Univariate analysis of fecal metabolites.** Boxplots were generated on the integrated and log transformed peak area extracted from the NMR spectrum. Pairwise comparison between elevated- and low-likelihood of ASD infants were investigated using Wilcoxon test: * *p* < 0.05, ** *p* < 0.01.


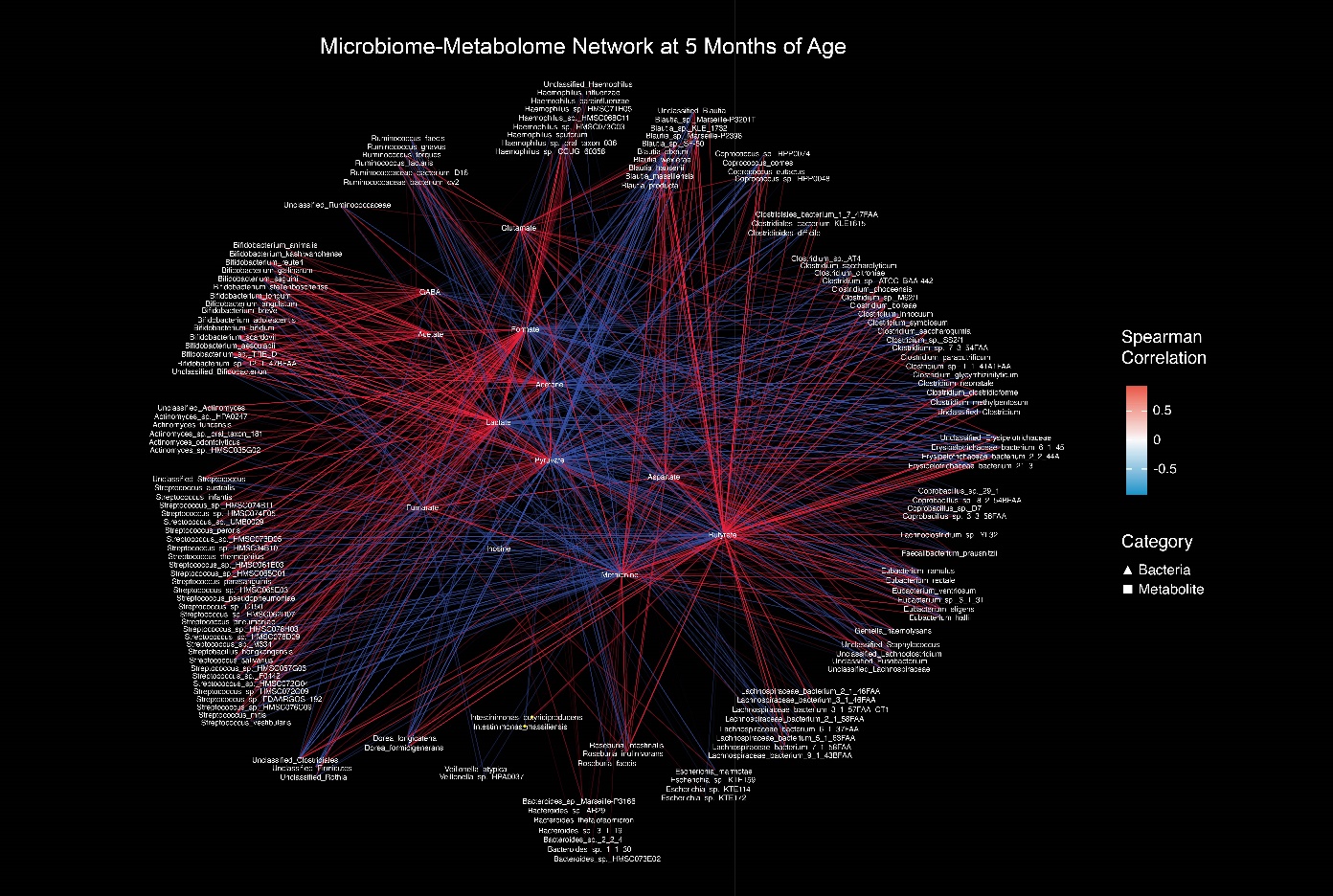


**Supplementary Figure 6. Microbiome-metabolome network at 5 months generated with Spearman’s correlations.** A correlation network was generated between CLR transformed OTU matrix and log transomed integrated metabolites matrix. Only correlation with FDR < 0.25 after BH correction were retained for visualization. Red edges indicate positive correlation while blue edges negative correlation.

**Supplementary Table 1. Summary of participant characteristics**

|  | **Infants at low-likelihood of ASD** | **Infants at elevated- likelihood of ASD** |
| --- | --- | --- |
| Infants n | 16 | 19 |
| Female sex | 10 | 9 |
| Maternal age (y) | 34.25 (± 4.420) | 35.54 (± 4.215) |
| Parental education^#^ |  |  |
| High school education | 3 | 3 |
| University education | 12 | 15 |
| Not reported | 1 | 1 |
| Family income per month* | 54,000 (± 15,020) | 54,440 (± 12,47) |

n, number

Data are mean ± SD

# Highest reported

*Amount in Swedish krona (SEK)

**Supplementary Table 2. List of OTUs correlation to PC1 and driving the time dependent development**.

| Family | Genus | Species | Corr PC1 |
| --- | --- | --- | --- |
| Alcaligenaceae | Achromobacter | Achromobacter_sp._ATCC35328 | -0.78 |
| Christensenellaceae | Christensenella | Christensenella_minuta | 0.71 |
| Clostridiaceae | Butyricicoccus | Butyricicoccus_pullicaecorum | 0.72 |
| Clostridiaceae | Clostridium | Clostridium_phoceensis | 0.81 |
| Clostridiaceae | Clostridium | Clostridium_sp._M62/1 | 0.82 |
| Clostridiaceae | Clostridium | Clostridium_sp._L2-50 | 0.77 |
| Clostridiaceae | Clostridium | Clostridium_sp._ATCC_29733 | 0.75 |
| Clostridiaceae | Massilioclostridium | Massilioclostridium_coli | 0.73 |
| Clostridiaceae | Mordavella | Mordavella_sp._Marseille-P3756 | 0.75 |
| Clostridiales_Family_XIII._Incertae_Sedis | Emergencia | Emergencia_timonensis | 0.74 |
| Enterobacteriaceae | Citrobacter | Unclassified_Citrobacter | -0.74 |
| Enterobacteriaceae | Citrobacter | Citrobacter_freundii | -0.81 |
| Enterobacteriaceae | Citrobacter | Citrobacter_braakii | -0.74 |
| Enterobacteriaceae | Citrobacter | Citrobacter_koseri | -0.78 |
| Enterobacteriaceae | Citrobacter | Citrobacter_sp._MGH103 | -0.73 |
| Enterobacteriaceae | Citrobacter | Citrobacter_rodentium | -0.79 |
| Enterobacteriaceae | Citrobacter | Citrobacter_sp._MGH_55 | -0.71 |
| Enterobacteriaceae | Citrobacter | Citrobacter_freundii_complex_sp._CFNIH2 | -0.72 |
| Enterobacteriaceae | Citrobacter | Citrobacter_freundii_complex_sp._CFNIH3 | -0.71 |
| Enterobacteriaceae | Citrobacter | Citrobacter_sp._MGH109 | -0.73 |
| Enterobacteriaceae | Citrobacter | Citrobacter_sp._MGH110 | -0.74 |
| Enterobacteriaceae | Cronobacter | Cronobacter_sakazakii | -0.79 |
| Enterobacteriaceae | Cronobacter | Unclassified_Cronobacter | -0.81 |
| Enterobacteriaceae | Enterobacter | Enterobacter_cloacae | -0.79 |
| Enterobacteriaceae | Enterobacter | Enterobacter_kobei | -0.78 |
| Enterobacteriaceae | Escherichia | Unclassified_Escherichia | -0.71 |
| Enterobacteriaceae | Escherichia | Escherichia_coli | -0.75 |
| Enterobacteriaceae | Escherichia | Escherichia_albertii | -0.79 |
| Enterobacteriaceae | Escherichia | Escherichia_sp._KTE172 | -0.76 |
| Enterobacteriaceae | Escherichia | Escherichia_fergusonii | -0.8 |
| Enterobacteriaceae | Escherichia | Escherichia_sp._1_1_43 | -0.81 |
| Enterobacteriaceae | Escherichia | Escherichia_sp._3_2_53FAA | -0.75 |
| Enterobacteriaceae | Escherichia | Escherichia_sp._KTE159 | -0.71 |
| Enterobacteriaceae | Escherichia | Escherichia_sp._KTE114 | -0.8 |
| Enterobacteriaceae | Escherichia | Escherichia_marmotae | -0.81 |
| Enterobacteriaceae | Escherichia | Escherichia_sp._KTE31 | -0.76 |
| Enterobacteriaceae | Klebsiella | Unclassified_Klebsiella | -0.79 |
| Enterobacteriaceae | Klebsiella | Klebsiella_michiganensis | -0.71 |
| Enterobacteriaceae | Klebsiella | Klebsiella_pneumoniae | -0.82 |
| Enterobacteriaceae | Klebsiella | Klebsiella_quasipneumoniae | -0.78 |
| Enterobacteriaceae | Klebsiella | Klebsiella_variicola | -0.74 |
| Enterobacteriaceae | Klebsiella | Klebsiella_aerogenes | -0.82 |
| Enterobacteriaceae | Klebsiella | Klebsiella_quasivariicola | -0.74 |
| Enterobacteriaceae | Klebsiella | Klebsiella_sp._A-Nf5 | -0.72 |
| Enterobacteriaceae | Klebsiella | Klebsiella_sp._M5al | -0.71 |
| Enterobacteriaceae | Klebsiella | Klebsiella_sp._HMSC25G12 | -0.71 |
| Enterobacteriaceae | Kluyvera | Kluyvera_cryocrescens | -0.8 |
| Enterobacteriaceae | Kosakonia | Kosakonia_radicincitans | -0.74 |
| Enterobacteriaceae | Pluralibacter | Pluralibacter_gergoviae | -0.73 |
| Enterobacteriaceae | Pseudescherichia | Pseudescherichia_vulneris | -0.73 |
| Enterobacteriaceae | Salmonella | Salmonella_enterica | -0.83 |
| Enterobacteriaceae | Shigella | Unclassified_Shigella | -0.78 |
| Enterobacteriaceae | Shigella | Shigella_sonnei | -0.8 |
| Enterobacteriaceae | Shigella | Shigella_flexneri | -0.8 |
| Enterobacteriaceae | Shigella | Shigella_dysenteriae | -0.81 |
| Enterobacteriaceae | Shigella | Shigella_boydii | -0.81 |
| Enterobacteriaceae | Trabulsiella | Trabulsiella_odontotermitis | -0.79 |
| Enterobacteriaceae | Unclassified_Enterobacteriaceae | Unclassified_Enterobacteriaceae | -0.83 |
| Enterobacteriaceae | Unclassified_Enterobacteriaceae | Enterobacteriaceae_bacterium_strain_FGI_57 | -0.78 |
| Enterobacteriaceae | Unclassified_Enterobacteriaceae | Enterobacteriaceae_bacterium_ENNIH1 | -0.75 |
| Enterobacteriaceae | Unclassified_Enterobacteriaceae | Enterobacteriaceae_bacterium_ENNIH2 | -0.72 |
| Enterococcaceae | Enterococcus | Unclassified_Enterococcus | -0.72 |
| Enterococcaceae | Enterococcus | Enterococcus_faecalis | -0.71 |
| Erwiniaceae | Erwinia | Erwinia_teleogrylli | -0.73 |
| Erysipelotrichaceae | Faecalitalea | Faecalitalea_cylindroides | 0.72 |
| Erysipelotrichaceae | Faecalitalea | Faecalitalea_sp._Marseille-P3755 | 0.71 |
| Erysipelotrichaceae | Holdemania | Holdemania_filiformis | 0.78 |
| Erysipelotrichaceae | Traorella | Traorella_massiliensis | 0.81 |
| Eubacteriaceae | Eubacterium | Eubacterium_eligens | 0.77 |
| Eubacteriaceae | Eubacterium | Eubacterium_hallii | 0.77 |
| Eubacteriaceae | Eubacterium | Eubacterium_ramulus | 0.72 |
| Eubacteriaceae | Eubacterium | Eubacterium_ventriosum | 0.8 |
| Lachnospiraceae | Anaerotignum | Anaerotignum_lactatifermentans | 0.72 |
| Lachnospiraceae | Blautia | Blautia_sp._Marseille-P3087 | 0.83 |
| Lachnospiraceae | Butyrivibrio | Butyrivibrio_crossotus | 0.85 |
| Lachnospiraceae | Coprococcus | Coprococcus_eutactus | 0.82 |
| Lachnospiraceae | Coprococcus | Coprococcus_comes | 0.76 |
| Lachnospiraceae | Dorea | Dorea_longicatena | 0.73 |
| Lachnospiraceae | Dorea | Dorea_formicigenerans | 0.74 |
| Lachnospiraceae | Fusicatenibacter | Fusicatenibacter_saccharivorans | 0.75 |
| Lachnospiraceae | Johnsonella | Johnsonella_ignava | 0.77 |
| Lachnospiraceae | Lachnoclostridium | Clostridium_saccharolyticum | 0.75 |
| Lachnospiraceae | Lachnospira | Lachnospira_pectinoschiza | 0.73 |
| Lachnospiraceae | Roseburia | Roseburia_intestinalis | 0.75 |
| Lachnospiraceae | Roseburia | Roseburia_inulinivorans | 0.82 |
| Lachnospiraceae | Roseburia | Roseburia_hominis | 0.8 |
| Lachnospiraceae | Roseburia | Unclassified_Roseburia | 0.82 |
| Lachnospiraceae | Unclassified_Lachnospiraceae | Eubacterium_rectale | 0.78 |
| Lachnospiraceae | Unclassified_Lachnospiraceae | Lachnospiraceae_bacterium_TF01-11 | 0.73 |
| Legionellaceae | Legionella | Legionella_pneumophila | -0.86 |
| Leptotrichiaceae | Streptobacillus | Streptobacillus_moniliformis | -0.84 |
| Leptotrichiaceae | Streptobacillus | Streptobacillus_hongkongensis | -0.77 |
| Morganellaceae | Xenorhabdus | Xenorhabdus_hominickii | -0.83 |
| Oscillospiraceae | Oscillibacter | Unclassified_Oscillibacter | 0.84 |
| Rikenellaceae | Alistipes | Alistipes_finegoldii | 0.77 |
| Rikenellaceae | Alistipes | Alistipes_putredinis | 0.74 |
| Rikenellaceae | Alistipes | Unclassified_Alistipes | 0.79 |
| Rikenellaceae | Alistipes | Alistipes_shahii | 0.76 |
| Rikenellaceae | Alistipes | Alistipes_onderdonkii | 0.76 |
| Rikenellaceae | Alistipes | Alistipes_sp._AL-1 | 0.71 |
| Rikenellaceae | Alistipes | Alistipes_senegalensis | 0.73 |
| Ruminococcaceae | Agathobaculum | Agathobaculum_desmolans | 0.75 |
| Ruminococcaceae | Anaeromassilibacillus | Anaeromassilibacillus_sp._Marseille-P3371 | 0.82 |
| Ruminococcaceae | Anaerotruncus | Anaerotruncus_colihominis | 0.79 |
| Ruminococcaceae | Angelakisella | Angelakisella_massiliensis | 0.85 |
| Ruminococcaceae | Faecalibacterium | Faecalibacterium_prausnitzii | 0.85 |
| Ruminococcaceae | Gemmiger | Gemmiger_formicilis | 0.78 |
| Ruminococcaceae | Ruminiclostridium | Eubacterium_siraeum | 0.76 |
| Ruminococcaceae | Ruminiclostridium | Clostridium_leptum | 0.75 |
| Ruminococcaceae | Ruminococcus | Ruminococcus_callidus | 0.76 |
| Ruminococcaceae | Ruminococcus | Ruminococcus_champanellensis | 0.72 |
| Ruminococcaceae | Ruminococcus | Ruminococcus_bromii | 0.71 |
| Ruminococcaceae | Subdoligranulum | Subdoligranulum_variabile | 0.79 |
| Ruminococcaceae | Unclassified_Ruminococcaceae | Unclassified_Ruminococcaceae | 0.73 |
| Ruminococcaceae | Unclassified_Ruminococcaceae | Ruminococcaceae_bacterium_D16 | 0.71 |
| Ruminococcaceae | Unclassified_Ruminococcaceae | Ruminococcaceae_bacterium_D5 | 0.76 |
| Staphylococcaceae | Staphylococcus | Staphylococcus_epidermidis | -0.77 |
| Streptococcaceae | Streptococcus | Streptococcus_sp._HMSC34B10 | -0.74 |
| Streptococcaceae | Streptococcus | Streptococcus_peroris | -0.74 |
| Unclassified_Clostridiales | Intestinimonas | Intestinimonas_butyriciproducens | 0.71 |
| Unclassified_Clostridiales | Pseudoflavonifractor | Pseudoflavonifractor_sp._Marseille-P3106 | 0.76 |
| Unclassified_Clostridiales | Pseudoflavonifractor | Pseudoflavonifractor_capillosus | 0.79 |
| Unclassified_Clostridiales | Unclassified_Clostridiales | Clostridiales_bacterium_KLE1615 | 0.76 |
| Unclassified_Clostridiales | Unclassified_Clostridiales | Bacteroides_pectinophilus | 0.83 |
| Unclassified_Enterobacterales | Unclassified_Enterobacterales | Unclassified_Enterobacterales | -0.86 |
| Veillonellaceae | Veillonella | Unclassified_Veillonella | -0.78 |
| Veillonellaceae | Veillonella | Veillonella_parvula | -0.73 |
| Veillonellaceae | Veillonella | Veillonella_sp._6_1_27 | -0.73 |
| Veillonellaceae | Veillonella | Veillonella_sp._3_1_44 | -0.74 |
| Veillonellaceae | Veillonella | Veillonella_rodentium | -0.79 |
